# Influence of time after extraction on the development of gingival invagination: study protocol for a multicenter pilot randomized controlled clinical trial

**DOI:** 10.1186/1745-6215-14-108

**Published:** 2013-04-24

**Authors:** Christoph Reichert, Eric Kutschera, Manuel Nienkemper, Sven Scharf, Martin Mengel, Rolf Fimmers, Christine Fuhrmann, Christina Plötz, Lina Gölz, Dieter Drescher, Bert Braumann, Andreas Jäger

**Affiliations:** 1 Department of Orthodontics, University Hospital Bonn, Welschnonnenstraße 17, 53111, Bonn, Germany; 2 Department of Orthodontics, University Hospital Düsseldorf, Moorenstraße 5, 40225, Düsseldorf, Germany; 3 Department of Orthodontics, University Hospital Cologne, Kerpener Straße 32, 50931, Köln, Germany; 4 Clinical Study Support Core, University Hospital Bonn, Sigmund-Freud-Straße 25, 53015, Bonn, Germany; 5 Institute for Medical Biometry, Informatics and Epidemiology Bonn, Sigmund-Freud-Straße 25, 53015, Bonn, Germany

**Keywords:** Gingival invagination, Tooth extraction, Orthodontic tooth movement, Space closure, Time, Clinical trial

## Abstract

**Background:**

Gingival invaginations are a common side effect of orthodontic therapy involving tooth extraction and subsequent space closure. Consequences of gingival invaginations are a jeopardized stability of the space closure and hampered oral hygiene. In a retrospective study, the factor time until initiation of orthodontic space closure after tooth extraction has been identified as a potential risk factor for the development of gingival invaginations. The aim of this pilot study is to proof this hypothesis and to enable a caseload calculation for further clinical trials. The referring question is: is it possible to reduce the number of developing gingival invaginations by initiation of orthodontic space closure after tooth extraction at an early point of time?

**Design:**

The intended pilot study is designed as a multicenter randomized controlled clinical trial, comparing the impact of two different time intervals from tooth extraction to initiation of orthodontic space closure on the development of gingival invaginations.

Forty participants, men and women in the age range of 11 to 30 years with orthodontically related indication for tooth extraction in the lower jaw, will be randomized 1:1 in one of two treatment groups. In group A the orthodontic tooth movement into the extraction area will be initiated in a time interval 2 to 4 weeks after tooth extraction. In group B the tooth movement will be initiated in a time interval >12 weeks after extraction. A possible effect of these treatment modalities on the development of gingival invaginations will be documented at the moment of space closure or 10 months +/- 14 days after initiation of space closure respectively, by clinical documentation of the primary (reduced number of gingival invagination) and the secondary endpoint (reduction of the severity of gingival invaginations).

**Trial registration:**

Universal Trial Number U1111-1132-6655; German Clinical Trials Register DRKS00004248

## Background

**Figure 1:**
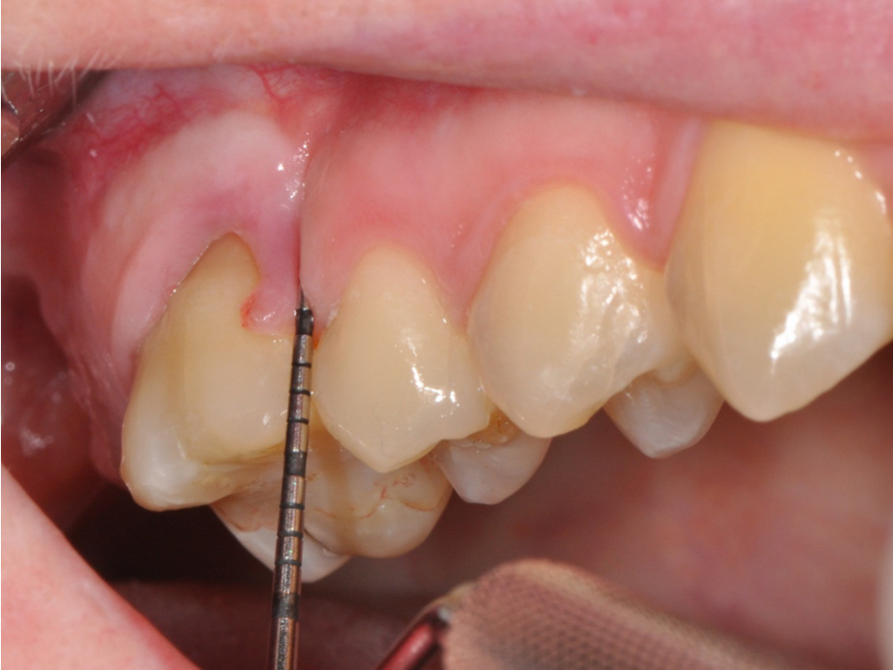
Exemplary illustration of a gingival invagination established after extraction of tooth 16 with subsequent orthodontic space closure.

Gingival invaginations are a common side effect of orthodontic therapy, involving tooth extraction and subsequent space closure (Figure 1) [1]. They are defined as a cleft of the alveolar process with vertical and horizontal probing depth of at least 1 mm, occurring after tooth extraction and subsequent orthodontic space closure [2]. Their incidence is given in the range of 30% to 100% [3,4]. Consequences of this side effect are marginal bone loss, hampered oral care, and jeopardized stability of the orthodontic treatment result [1].

In retrospective studies several potential risk factors for the development of gingival invaginations were evaluated, namely time from tooth extraction up to initiation of tooth movement, localization of the extraction area, smoking and duration of space closure [2,3,5]. Further, data of an animal experiment [6] suggested that early tooth movement into an extraction site could be beneficial to prevent gingival invaginations, so atrophy of the alveolar process does not proceed.

In this clinical study we try to prove the hypothesis that a timely tooth movement into an extraction area could be beneficial and prevent the development of gingival invaginations.

## Methods/Design

### Study objectives

The objective of the clinical study proposal will be to investigate the effects of a timely *versus* a prolonged space closure after tooth extraction on the incidence and severity of gingival invaginations. The study will compare two treatment groups: in group A the orthodontic tooth movement into the extraction area will be initiated in a time interval 2 to 4 weeks after tooth extraction. In group B the tooth movement will be initiated in a time interval >12 weeks after extraction. The null hypothesis will be addressed: There will be no difference of both treatment modalities on the incidence or the severity of gingival invaginations.

### Trial design

**Figure 2:**
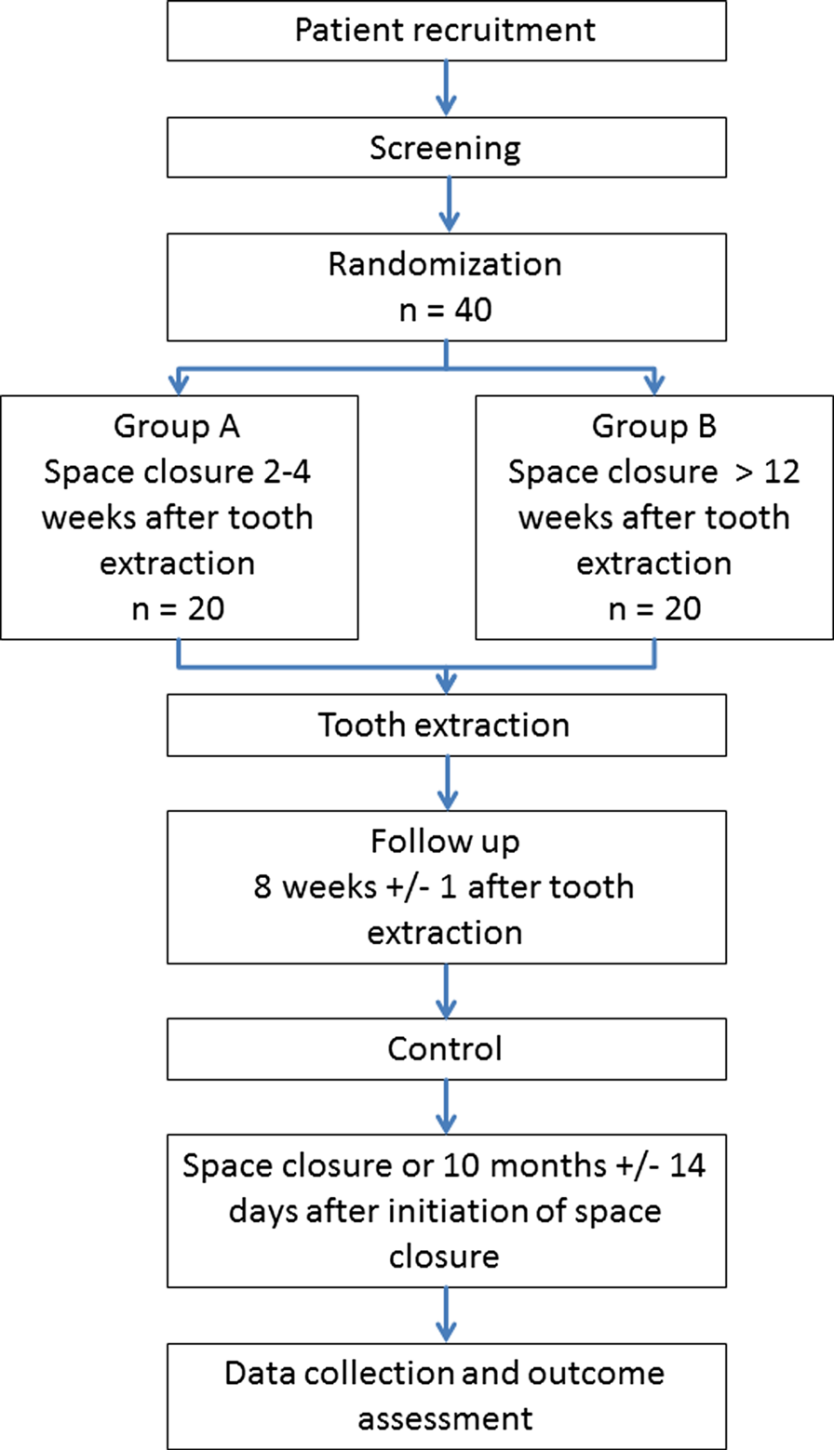
**Work flow for the study.** After recruitment and screening the patients will be randomized in one of the two groups. After the tooth extraction the patients will be treated equally, but space closure will be initiated at different time points.

The study is designed as a multicenter pilot randomized controlled clinical trial (RCT). The study was approved by the Ethics Committee of the University Bonn, Germany (Ref. No. 061/12), Cologne, Germany (Ref. No. 12-210) and Düsseldorf, Germany (3979). A formal coordination center for clinical trials (CSSC, Bonn, Germany) will monitor the study process and assure data accuracy. Data management will be performed by an Institute of Medical Biostatistics (Institute for Medical Biometry, Informatics and Epidemiology, IMBIE, Bonn, Germany). A flow chart for the study is given in Figure 2.

### Participants

Men and women whose diagnoses require tooth extraction in the lower jaw will be included in the study. Inclusion criteria are as follows:

• Orthodontically related indication for extraction of at least one premolar in the lower jaw

• Age ≥11 years up to and including 30 years

• Informed consent in words and written by the patient and his or her legal guardian

• Patient’s ability to follow the advices and to participate in the study visits

Patients with syndromes, diseases, or medication with strong impact on bone and connective tissue metabolism as well as non-compliance to the orthodontic therapy are excluded from the study (for example, medication with bisphosphonates or interferon or syndromes like cleidocranial dysplasia). A further exclusion criterion will be complications during the tooth extraction or wound healing, such as fracture of the alveolar bone, the root, or an infection during wound healing. In this case not the patient, but the extraction region will be excluded from the study.

### Study interventions

The study intervention will be: the initiation of the orthodontic tooth movement in a time interval 2 to 4 weeks after tooth extraction (group A) *versus* tooth movement initiated in a time interval >12 weeks after extraction (group B). Once the space closure started it should be continued regardless of the applied mechanics up to the endpoint ‘space closure’ or the endpoint ‘10 months +/- 14 days after initiation of the space closure’. Space closure is defined as binding of dental floss in the contact point of the neighboring teeth in the former extraction region.

### Screening

All patients seeking orthodontic treatment in one of the study centers are potential candidates to participate in this study. After planning the treatment the coordinator is checking the inclusion and exclusion criteria. If all criteria are fulfilled the patient will be informed about the study. Given consent to participate, the patient will be randomized.

### Sample size

The sample size is set on 20 patients per group. This sample size is not primary designated by statistical considerations. The aim of this pilot study is to gain first perceptions about behavior and distribution of study-related parameters. Obtaining data should enable a caseload calculation for future clinical trials. Possible results of a power analysis involving Fisher’s exact test comparing the incidence of gingival invaginations in both groups is given in Table 1. The differences do not reflect expected effects with both therapies; they rather demonstrate varieties which might be revealed in the chosen sample size.

**Table 1:** Power for 2×20 patients calculated with a two-sided Fisher’s exact test

**p1**	**p2**				
	**0.8**	**0.75**	**0.7**	**0.65**	**0.6**
1.00	37.0%	58.5%	76.2%	88.2%	94.9%
0.95	-	29.9%	44.6%	58.9%	71.6%
0.90	-	-	24.2%	36.1%	49.2%

### Randomization

Randomization is stratified by center and organized as fax-randomization. After inclusion of a patient into the study a fax containing the screening number is sent to the coordinating center, where randomization is performed according to prepared lists for each center. The reply, containing randomization number and treatment, is also sent by fax.

### Tooth extraction

After randomization, the patient will be referred to an oral surgeon of the study centers. Here the designated teeth will be extracted. Extraction technique, anesthesia used, and eventual complications during extraction like root fracture or flap elevation will be documented. Further, a replica of the roots of the extracted teeth will be established. Therefore the apex will be impressed in long axis into putty material for a possible replication with plaster.

### Initiation of the space closure

At this point, a plaque-index [7], a bleeding index [8], probing depth of the adjacent teeth according to the Periodontal Screening Index (PSI) [9], stone models, and intraoral photos will be gathered. The coordinator of each center will activate and document the mechanic for space closure.

### Follow-up and control

During regular treatment, remaining extraction space as well as a possible adaptation of the treatment mechanics will be documented at the regular recall visits, every 4 to 6 weeks.

Eight weeks +/- 1 after initiation of the space closure another stone model and intraoral photos will be gathered.

### Endpoint - space closure or 10 months +/- 14 days after initiation of space closure

The primary end point of the study is the fulfilled space closure. If the time for space closure of a patient included in our study would exceed 10 months, this could possibly reveal problems in the treatment process. As a consequence an alternative end point was set on 10 months +/- 14 days after initiation of space closure.

This appointment obtains another plaque- and bleeding index, probing depth of the adjacent teeth, stone models, and intraoral photos. Further, the fulfilled space closure, the occurrence of gingival invaginations, their dimension and time from initiation up to space closure will be recorded.

### Outcome measurements

#### Primary outcome measurement

Primary outcome measurement will be a determination of the number of gingival invaginations at the moment of space closure. A gingival invagination is present, if there is a probing depth of at least 1 mm parallel to the occlusal plain in vestibular or oral orientation measured 2 mm caudal from the crest ‘free gingival margin to junctional epithelium’.

#### Secondary outcome measurement

Secondary endpoint will be the dimension according to LC^2^[2]. If a gingival invagination is present its entire depth will be examined using a periodontal probe (Periodontmeter from Williams 17; Hu-Fridey, Rotterdam, The Netherlands). The measurement will be taken parallel to the occlusal plane in vestibular and oral orientation and in vertical direction at a 90° angle to the occlusal plane. Maximum values from all three angles will be noted in a three-figure code, for example, probes of 3 mm in a vestibular orientation, 2 mm vertical, and 4 mm oral result in the code: 3-2-4. Complete penetration in oro-/vestibular orientation will be identified with an x and only the vertical penetration will be noted (for example, ×4).

### Blinding

To ensure quality and neutrality of the obtained data a blinded rater will perform the documentation at the time points ‘Initiation of the space closure’ and ‘End of the study’.

### Statistical methods

Primary aim of the study is the estimation of the frequency of gingival invaginations under both therapies. In group A the initiation of the orthodontic tooth movement will be performed in a time interval 2 to 4 weeks after tooth extraction *versus* tooth movement initiated in a time interval >12 weeks after extraction (group B). At the endpoints ‘space closure’ or ‘10 months +/- 14 days after initiation of the space closure’, the frequencies of gingival invaginations will be given together with 95% confidence limits. Also the odds ratio for the occurrence of gingival invaginations in both study arms will be calculated together with a 95% confidence interval. Finally, a two-sided Fisher’s exact test will be performed to descriptively compare the rates between treatment arms.

For the secondary aim, the difference in the dimensions of the gingival invaginations according to LC^2^, the median, minimum and maximum values as well as quartiles will be given. For the comparison of the values a two-sided Mann-Whitney-Wilcoxon test will be performed. Also the odds ratio in both study arms will be calculated together with a 95% confidence interval. In the case of an absence of a gingival invagination LC^2^ will be set to zero.

If some patients do not achieve the primary endpoint ‘fulfilled space closure’ and finish the study within the alternative endpoint ‘10 months +/- 14 days after initiation of space closure’ we would excluded these patients for later statistical analysis because they could possibly represent confounders.

## Discussion

This study is a preliminary study targeting on the effect of a timely coordinated space closure after tooth extraction on the development of gingival invaginations. Other factors like extraction technique, treatment mechanics for space closure or patient-related features could also have an impact on their development. As there is no prospective data up to now, this study can only have preliminary character and therefore it is focused on only one potential risk factor. Having this data, it will be possible to plan future RCTs with adequate sample size, focusing on new risk factors identified in this study.

## Trial status

Patient recruitment since 10.12.2012

## Abbreviations

Ref. No.: Reference number; CSSC: Clinical study support core; IMBIE: Institute for Medical Biometry, Informatics and Epidemiology; RCT: Randomized controlled trial.

## Competing interests

The authors declare that they have no competing interests.

## Authors’ contributions

CR, AJ and MM designed the study.CR, MN, EK and SS are the main contact people at the study centers. CP and LG are responsible for data collection. RF is responsible for sample size calculation, data management and biometrical support. DD, BB and AJ provide the infrastructure. They have international reputation in biomechanics and their experience will be provided for the investigators (CR, MN, SS, EK, LG, CP). MM and CF will monitor the study. They are responsible for logistic, communication with the ethics committee, and coordination between study centers and biometry. All authors read and approved the final manuscript.
